# Evaluation of Right Ventricular Myocardial Mechanics by 2- and 3-Dimensional Speckle-Tracking Echocardiography in Patients With an Ischemic or Non-ischemic Etiology of End-Stage Heart Failure

**DOI:** 10.3389/fcvm.2022.765191

**Published:** 2022-05-25

**Authors:** Fangyan Tian, Ying Gu, Yanting Zhang, Bei Zhang, Yuji Xie, Shaomei Yu, Shuangshuang Zhu, Wei Sun, Shan Cheng, Mingzu Qian, Yixia Lin, Wenqian Wu, Yali Yang, Qing Lv, Jing Wang, Li Zhang, Yuman Li, Mingxing Xie

**Affiliations:** ^1^Department of Ultrasound, Union Hospital, Tongji Medical College, Huazhong University of Science and Technology, Wuhan, China; ^2^Clinical Research Center for Medical Imaging in Hubei Province, Wuhan, China; ^3^Hubei Province Key Laboratory of Molecular Imaging, Wuhan, China; ^4^Department of Ultrasound Medicine, The Affiliated Hospital of Guizhou Medical University, Guiyang, China

**Keywords:** three-dimensional, two-dimensional, speckle tracking echocardiography, right ventricular function, strain, heart failure

## Abstract

**Background:**

The aims of our study were (1) to assess the right ventricular (RV) myocardial mechanics by two-dimensional (2D) and three-dimensional (3D) speckle-tracking echocardiography (STE) in patients with an ischemic or non-ischemic etiology of end-stage heart failure (HF) and (2) to explore which RV index evaluated by 2D- and 3D-STE was the most powerful indicator for identifying the ischemic and non-ischemic etiologies of end-stage HF.

**Methods:**

A total of 96 patients with left ventricular ejection fraction (LVEF) < 30% were enrolled in our study: 42 patients (mean age, 52 ± 10 years; 9.5% female) with ischemic cardiomyopathy and 54 patients (mean age, 46 ± 14 years; 16.7% female) with non-ischemic cardiomyopathy. A total of 45 healthy subjects (mean age, 46 ± 13 years; 24.4% female) served as controls. The longitudinal strain of the RV free wall (RVFWLS) was determined by both 2D- and 3D-STE.

**Results:**

Compared to controls, patients with an ischemic or non-ischemic etiology of end-stage HF had lower 2D-RVFWLS, 3D-RVFWLS and RV ejection fraction (RVEF) values (*P* < 0.05). Patients with non-ischemic cardiomyopathies (NICMs) had significantly lower 3D-RVFWLS and RVEF values than in those with ischemic cardiomyopathies (ICMs), whereas 2D-RVFWLS and conventional RV function parameters did not differ between the two subgroups. RVEF was highly related to 3D-RVFWLS (*r* = 0.72, *P* < 0.001), modestly related to 2D-RVFWLS (*r* = 0.51, *P* < 0.001), and weakly related to conventional RV function indices (*r* = –0.26 to 0.46, *P* < 0.05). Receiver operating characteristic curve analysis revealed that the optimal 3D-RVFWLS cut-off value to distinguish NICM from ICM patients was –14.78% (area under the curve: 0.73, *P* < 0.001), while 2D-RVFWLS and conventional RV echocardiographic parameters did not.

**Conclusion:**

Our study demonstrated the superiority of 3D-RVFWLS over 2D-RVFWLS and conventional RV function indices in identifying the ischemic and non-ischemic etiologies of end-stage HF. These findings support the idea that 3D-RVFWLS may be a promising non-invasive imaging marker for distinguishing NICM from ICM.

## Introduction

Right ventricular (RV) function has a powerful capability for risk-stratifying patients with heart failure (HF) (1–3). In these patients, indeed, the presence of RV dysfunction is associated with adverse outcomes (4, 5). Non-ischemic cardiomyopathies (NICMs) are the most frequent cause of HF and death, and patients with NICMs have a poorer outcome than those with ischemic cardiomyopathies (ICMs) (6). For this reason, accurately distinguishing the non-ischemic and ischemic etiologies of HF is of great clinical significance. Coronary angiography, as the gold-standard modality for diagnosing ICM, is not used in every case due to its invasiveness, requirement of ionizing radiation, and high cost (7). Therefore, it is crucial to explore other non-invasive parameters to discern between the ischemic and non-ischemic etiologies of HF.

Echocardiography, which is readily available and relatively inexpensive, is considered a first-line modality for assessing ventricular performance. Nevertheless, completing an accurate assessment of RV function by traditional echocardiography is challenging because of its complex structure, contraction pattern, and response to overload. Two-dimensional (2D) speckle-tracking echocardiography (STE) is an angle-independent, less load-dependent technique that allows for the earlier identification of subtle RV dysfunction. It has been demonstrated to be a more reliable and accurate tool for RV function assessment than conventional RV function indices (8–10). However, this algorithm, based on the 2D plane, has limitations, which lead to the loss of a portion of the real motion due to out-of-plane motion (11). Recently, three-dimensional (3D) STE has emerged to overcome these limitations (12). Its accuracy and reproducibility in quantifying RV function have been verified in patients with pulmonary hypertension, transplanted hearts, and hypoplastic left heart syndrome after Fontan palliation (13–15). However, the possibility of interrogating RV mechanics in patients with end-stage HF using 3D-STE has hitherto not been explored.

Thus, the purposes of our study were (1) to assess RV myocardial mechanics using 2D- and 3D-STE in subjects with an ischemic or non-ischemic etiology of end-stage HF and (2) to explore which RV index evaluated by 2D- and 3D-STE has the potential to differentiate between the ischemic and non-ischemic etiologies of end-stage HF.

## Materials and Methods

### Study Subjects

This was a prospective study of 109 consecutive patients with end-stage HF who required heart transplantation (HT) and were enrolled in this study at the Wuhan Union Hospital from June 2018 to July 2021. All patients had severely impaired left ventricular (LV) function [LV ejection fraction (LVEF) < 30%] (16), and their New York Heart Association (NYHA) functional class was III or IV. Patients were assigned to the ICM group if they had a prior history of myocardial infarction/revascularization and/or if they had significant coronary artery stenosis (≥50%) in ≥ 1 epicardial coronary vessel on angiography. Patients were classified as having NICM if they had none of these features (17). Exclusion criteria included an anomalous origin of the coronary artery, cardiac arrhythmia, and poor echocardiographic image quality.

Separately, we enrolled a control group of 45 healthy volunteers with a similar age and sex breakdown with no cardiovascular disease on the basis of clinical examination, electrocardiography, echocardiography, and chest X-ray imaging. This study was approved by the ethics committee of Tongji Medical College, Huazhong University of Science and Technology, and written informed consent was obtained from all participants.

### Standard Echocardiography

All patients underwent transthoracic echocardiograms using the Philips EPIQ7C ultrasound machine (Philips Medical Systems, Andover, MA, United States). LV and RV parameters were measured according to the guidelines of the American Society of Echocardiography (18). The RV base diameter (RVD1), mid-diameter (RVD2), and length diameters (RVD3) were acquired from the RV-focused apical 4-chamber view. The RV fractional area change (FAC) was defined as the RV end-diastolic area – RV end-systolic area)/end-diastolic area × 100%. The tricuspid annular peak systolic excursion (TAPSE) was measured using M-mode echocardiography. The right-side index of myocardial performance (RIMP) was determined using tissue Doppler imaging. Peak systolic (s’) tricuspid lateral annular velocities were also assessed by tissue Doppler imaging. The apical 4-chamber view was used for 2D-STE analysis, and 3D full-volume data were obtained from the apical 4-chamber view with four consecutive cardiac cycles.

### Speckle-Tracking Echocardiography

**FIGURE 1:**
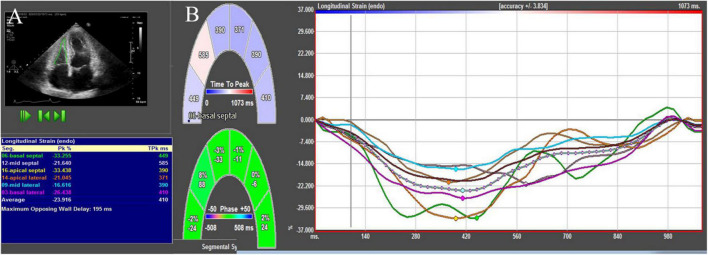
2D-STE showing RV endocardial border and the longitudinal strain values **(A)** and longitudinal strain curves **(B)**.

**FIGURE 2:**
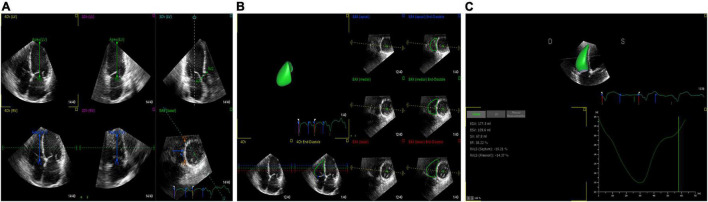
3D-STE offline analysis. **(A)** Setting reference points. **(B)** RV endocardial border identification and tracking. **(C)** Generating longitudinal strain of the RV free wall and RV volume curve.

2D-STE and 3D-STE analyses were performed using commercial software (4D-RV Function Analysis and 4D-LV Analysis version 3.1 software for 3D-STE and 2D Cardiac Performance Analysis for 2D-STE; Tom Tec Imaging Systems, Munich, Germany). For 2D-STE analysis, RV endocardial tracings were manually performed in the apical 4-chamber view. The software automatically tracked the speckle patterns in the myocardium. The endocardial border could be manually modified if necessary. Ultimately, the software generated the longitudinal strain curves and longitudinal peak systolic strain values of 6 segments of the RV. The 2D longitudinal strain of the RV free wall (2D-RVFWLS) was defined as the mean value of three segments of the RV free wall (Figure 1). For 3D-STE analysis, the operator set reference points (i.e., the center of the mitral annular line to the apex of the LV, the center of the tricuspid annular line to the apex of the RV, landmarks corresponding to the aortic annulus diameter, and the anterior and posterior junctions of the RV free wall with the interventricular septum and the septum-to-RV free wall) in 6 planes. Subsequently, the RV endocardial border was automatically identified by the software. Then, the software automatically tracked the RV endocardial border throughout the cardiac cycle, although the operator could manually adjust the RV contours if necessary. Finally, the software produced the RV end-diastolic volume (RVEDV), RV end-systolic volume (RVESV), RV stroke volume (RVSV), RV ejection fraction (RVEF), and 3D longitudinal strain values of the RV free wall (3D-RVFWLS) (Figure 2). We determined the RVEDV index (RVEDVI), RVESV index (RVESVI) normalized to BSA. Similarly, the operator set reference points (the center of the mitral annular line to the apex of the LV) in the apical 4-, 2-, and 3-chamber views. Then, the LV endocardial border was automatically identified by the software, and a manual adjustment was performed if necessary. Finally, the LVEDVI (ml/m^2^ = LVEDV/BSA), LVESVI (ml/m^2^ = LVESV/BSA), LV mass index (LVMI) (g/m^2^ = LVM/BSA), and LV global longitudinal strain (LVGLS) were obtained. The frame rate range of the 3D STE images was set at approximately 20 Hz or more.

### Cardiac Magnetic Resonance Imaging Analysis

A total of 28 patients underwent cardiac magnetic resonance (CMR) examinations to assess RVEF within 1 day of echocardiography since CMR was regarded as the gold standard for RV systolic function. CMR imaging was analyzed using conventional CMR software (Argus; Siemens Medical Solutions, Erlangen, Germany). RV endocardial contours were manually traced on all short-axis slices on the end-diastolic and end-systolic frames by an experienced operator who was blinded to echocardiographic measurements. Finally, the software automatically obtained the CMR-RVEF.

### Right Heart Catheterization

All patients underwent right heart catheterization before HT. A Swan-Ganz catheter were used to acquire cardiac hemodynamic data. Right atrial pressure and pulmonary artery pressure were obtained from right heart catheterization.

### Statistical Analysis

Continuous variables are expressed as mean ± standard deviation values, non-normal distribution of continuous data are expressed as median (IQR),and categorical variables are expressed using frequency (percentage) values. Statistical significance was assessed by 1-way ANOVA followed by the Bonferroni *post hoc* test or by Student’s t-test when only two groups were compared. For non-normally distributed data, Man-Whitney U test and Kruskal-Wallis test were used. Logistic regression analysis was used to evaluate the effect of explanatory variables. To calculate the sensitivity and specificity at various cutoff levels for the selected parameters, receiver operating characteristic (ROC) curves were used. Pearson’s correlation coefficient was used for the assessment of correlations. Bland–Altman analysis and the intraclass correlation coefficient (ICC) to assess the consistency between 2D- and 3D-STE parameters were applied (19). The SPSS version 22.0 software (IBM Corporation, Armonk, NY, United States) and MedCalc version 19.0.4 software (MedCalc Software, Ostend, Belgium) were used for statistical analyses. A *P* value of < 0.05 in a two-sided test was considered to be statistically significant.

### Reproducibility Analysis

The intra- and inter-observer variability of 2D- and 3D-STE parameters were evaluated by Bland–Altman analysis and the ICC. To assess the reproducibility, 20 patients were randomly selected from our study. For the assessment of intra-observer variability, the data were re-analyzed by the same investigator after 1 month. For the evaluation of inter-observer variability, the second investigator re-analyzed the data while blinded to the values obtained by the first investigator.

## Results

### Clinical and Echocardiographic Characteristics

Following the exclusion of five patients with poor image quality, two with one-vessel coronary artery disease, and six with cardiac arrhythmias, 96 patients were included in our analysis. Of these 96 patients, 42 had ICM and 54 had NICM. The mean age of patients with end-stage HF was 48 ± 13 years, and 83 (86.5%) were men. Eighty (83.8%) patients had NYHA functional class IV. Tricuspid regurgitation (TR) was absent in 22 (22.9%), mild in 29 (30.2%), moderate in 17 (17.7%), and severe in 28 (29.2%) patients with end-stage HF, respectively. The clinical and echocardiographic data of the study participants are listed in Table 1. Sex, age, body surface area, prevalence of hypertension and diabetes, NYHA functional class, medical therapy, proportion of implantable cardioverter-defibrillator/cardiac resynchronization therapy-defibrillator (ICD/CRT-D) and laboratory data were not statistically different among the three groups. Patients with NICM had lower systolic and diastolic blood pressure values compared to the ICM and controls (*P* < 0.001), although they remained within the normal ranges. Compared to the controls, patients with ICM and NICM had increased RVEDVI, RVESVI, RVD1, RVD2, RVD3, RIMP, LVEDVI, LVESVI and LVMI values (*P* < 0.05), and decreased LVEF, LVGLS, TAPSE, RVFAC, tricuspid s’, RVEF, 2D-RVFWLS, and 3D-RVFWLS values (*P* < 0.05). Patients with NICM had decreased 3D-RVFWLS and RVEF values, and increased RVEDVI and RVESVI compared to those with ICM (*P* < 0.05); however, no significant differences in LVEDVI, LVESVI, LVMI, LVEF, RVD1, RVD2, RVD3, conventional RV function parameters (TAPSE, RVFAC, tricuspid s’, and RIMP), LVGLS and 2D-RVFWLS between the two subgroups were identified in our study. The severity of TR was similar between the ICM and NICM subgroups. CMR and right heart catheterization data were not significantly different between the groups (Table 2). In addition, we compared RVFWLS between patients with and without ICD/CRT-D, and our results revealed that 3D-RVFWLS and 2D-RVFWLS values did not differ between patients with and without ICD/CRT-D.

**TABLE 1:** Clinical and echocardiographic characteristics of patients and normal controls.

Variables	Controls (*n* = 45)	ICM(*n* = 42)	NICM (*n* = 54)	*p*
Female (%)	11 (24.4%)	4 (9.5%)	9 (16.7%)	0.141
Age (years)	46 ± 13	52 ± 10	46 ± 14	0.052
Body surface area (m^2^)	1.64 ± 0.40	1.73 ± 0.19	1.66 ± 0.33	0.582
Systolic blood pressure (mmHg)	116 ± 7	116 ± 21	101 ± 19*^†^	<0.001
Diastolic blood pressure (mmHg)	75 ± 8	74 ± 18	66 ± 12*^†^	<0.001
Hypertension		19 (45.2%)	15 (27.8%)	0.076
Diabetes mellitus		14 (33.3%)	9 (16.7%)	0.058
**NYHA functional class, *n* (%)**				0.581
III		6 (14.3%)	10 (18.5%)	
IV		36 (85.7%)	44 (81.5%)	
**Medical therapy (*n*, %)**				
Beta-blockers		42 (100%)	54 (100%)	
ACE inhibitors/ARBs		42 (100%)	54 (100%)	
Loop diuretics		41 (97.6%)	52 (96.3%)	>0.999
Aldosterone antagonists		40 (95.2%)	51 (94.4%)	>0.999
ICD/CRT-D		3 (7.1%)	9 (16.7%)	>0.999
**Laboratory data**				
Hemoglobin (g/L)		124.00 ± 17.78	133.46 ± 23.25	0.085
Total cholesterol (mmol/L)		3.03 (2.19, 3.52)	3.12 (2.63, 3.86)	0.357
Triglyceride (mmol/L)		1.37 (1.02, 2.13)	1.07 (0.81, 1.84)	0.715
Creatinine (μmol/L)		88 (78.2, 103.6)	85.8 (70.4, 105.85)	0.380
**Echocardiography**				
LVEDVI (ml/m^2^)	48 (40, 56)^∧^^†^	124 (91,141)	143 (120, 167)	<0.001
LVESVI (ml/m^2^)	15 (12,19)^∧^^†^	95 (77, 108)	105 (84, 135)	<0.001
LVEF (%)	67.51 ± 4.41^∧^^†^	25.19 ± 6.39	24.54 ± 6.06	<0.001
LVMI (g/m^2^)	86 (73, 92)^∧^^†^	177 (169, 216)	203 (185, 249)	<0.001
LVGLS (%)	–21.92 ± 2.59^∧^^†^	–6.16 ± 2.05	–7.46 ± 2.17	<0.001
RVD1 (mm)	27.51 ± 4.32^∧^^†^	36.24 ± 6.83	36.94 ± 9.60	<0.001
RVD2 (mm)	27.93 ± 4.05^∧^^†^	32.69 ± 8.17	32.66 ± 9.60	<0.001
RVD3 (mm)	66.76 ± 8.04^∧^^†^	76.48 ± 9.76	80.91 ± 12.27	<0.001
Mild TR (*n*, %)		15 (33.3%)	14 (25.5%)	0.388
Moderate TR (*n*, %)		5 (11.1%)	12 (21.8%)	0.156
Severe TR (*n*, %)		9 (20%)	19 (35.2%)	0.095
TAPSE (mm)	21.48 ± 2.76^∧^^†^	12.76 ± 2.46	11.99 ± 2.40	<0.001
RVFAC (%)	46.87 ± 4.67^∧^^†^	27.25 ± 5.79	25.03 ± 5.63	<0.001
RIMP	0.36 ± 0.03^∧^^†^	0.59 ± 0.05	0.67 ± 0.03	<0.001
Tricuspid s’ (cm/s)	12.84 ± 1.98^∧^^†^	9.05 ± 2.74	9.90 ± 2.66	<0.001
2D-RVFWLS (%)	–23.38 ± 8.90^∧^^†^	–14.29 ± 4.90	–13.24 ± 3.49	<0.001
RVEDVI (ml/m^2^)	47 (39,57)^∧^^†^	65 (50,76)	76 (53,100) *	<0.001
RVESVI (ml/m^2^)	26 (21,29)^∧^^†^	46 (32,53)	53 (38,72) *	<0.001
RVSV (ml)	44 (33,55)^∧^^†^	38 (24,45)	34 (24,42)	0.002
RVEF (%)	53.14 ± 4.49^∧^^†^	31.87 ± 9.68	28.09 ± 6.87*	<0.001
3D-RVFWLS (%)	–23.78 ± 2.15^∧^^†^	–15.36 ± 4.76	–11.92 ± 2.81*	<0.001

*Data are mean ± SD, n (%), or median (IQR).*

*P < 0.05 for ICM vs. NICM.

*^∧^ P < 0.05 for ICM vs. controls.*

*^†^P < 0.05 for NICM vs. controls.*

*ACE, angiotensin-converting enzyme; ARB, angiotensin receptor blocker; ICD, Implantable cardioverter defibrillator; CRT-D, cardiac resynchronization therapy-defibrillation; RV, right ventricular; LV, left ventricular; MI, mass index; EDVI, end-diastolic volume index; ESVI, end-systolic volume index; SV, stroke volume; EF, ejection fraction; RVD1, right ventricular basal diameter; RVD2, right ventricular mid diameter; RVD3, right ventricular longitudinal dimension; TR, tricuspid regurgitation; TAPSE, tricuspid annular plane systolic excursion; FAC, fractional area change; RIMP, right-side index of myocardial performance; Tricuspid s’, tricuspid annulus systolic velocity; 3D, three dimensional; 2D, two dimensional; RVFWLS, right ventricular free wall longitudinal strain.*

**TABLE 2:** Cardiac magnetic resonance and right heart catheterization characteristics of patients.

Variables	ICM (*n* = 42)	NICM (*n* = 54)	*p*
**Cardiac magnetic resonance**			
CMR-RVEF (%) (*n*)	14 (11,31) (8)	15 (11,22) (20)	0.739
**Right heart catheterization**			
Systolic PAP (mmHg)	45 ± 16	42 ± 15	0.313
Diastolic PAP (mmHg)	75 ± 18	66 ± 12	0.852
Mean PAP (mmHg)	30 ± 11	28 ± 12	0.591
RAP (mmHg)	10 (7,13)	9 (7,12)	0.780

*Data are mean ± SD or median (IQR). CMR-RVEF, cardiac magnetic resonance-right ventricular ejection fraction; PAP, pulmonary arterial pressure; RAP, right atrial pressure.*

**FIGURE 3:**
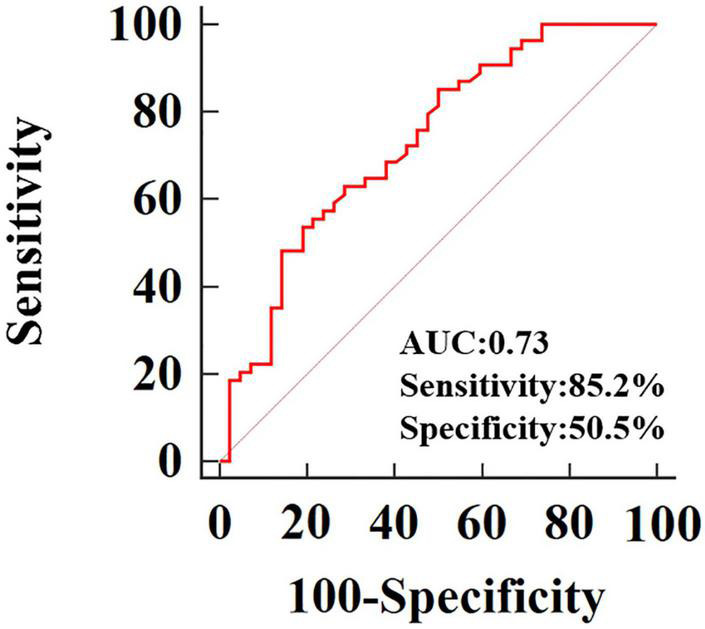
Receiver operating characteristic curves of 3D-RVFWLS for distinguishing NICM from ICM.

After adjustment for age, systolic and diastolic blood pressure, RVEDVI, RVESVI, RVEF, only 3D-RVFWLS remained significantly associated with NICM (odds ratio: 0.79; 95%CI:0.684–0.912; *P* = 0.001). 3D-RVFWLS, 2D-RVFWLS, LVGLS, and conventional RV function echocardiographic parameters were entered into ROC analysis to distinguish NICM and ICM patients from each other. ROC analysis revealed that the optimal cutoff value of 3D-RVFWLS was –14.78%, with a sensitivity of 85.2% and a specificity of 50.5%, for distinguishing between the ischemic and non-ischemic etiologies of end-stage HF (area under the ROC curve, 0.73; 95% confidence interval, 0.63–0.82; *P* < 0.001) (Figure 3). 2D-RVFWLS, LVGLS, and conventional RV echocardiographic parameters failed to distinguish NICM patients from ICM patients.

**FIGURE 4:**
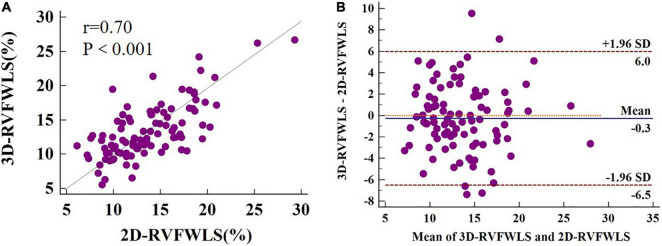
2D-3D longitudinal strain of RV free wall correlation plots **(A)** and Bland–Altman plots **(B)**. 3D-RVFWLS and 2D-RVFWLS values are absolute values.

Figure 4 presents Bland–Altman analysis and correlation plots for RVFWLS as measured by 2D- and 3D-STE. 3D-RVFWLS was strongly related to 2D-RVFWLS (*r* = 0.70, *P* < 0.001). Good consistency for RVFWLS as assessed by 2D- and 3D-STE, respectively, was noted (ICC, 0.70; 95% CI, 0.59–0.79).

### Relationships Between RV Ejection Fraction, Two-Dimensional- and Three-Dimensional-Speckle-Tracking Echocardiography, and Conventional RV Echocardiographic Indices

**FIGURE 5:**
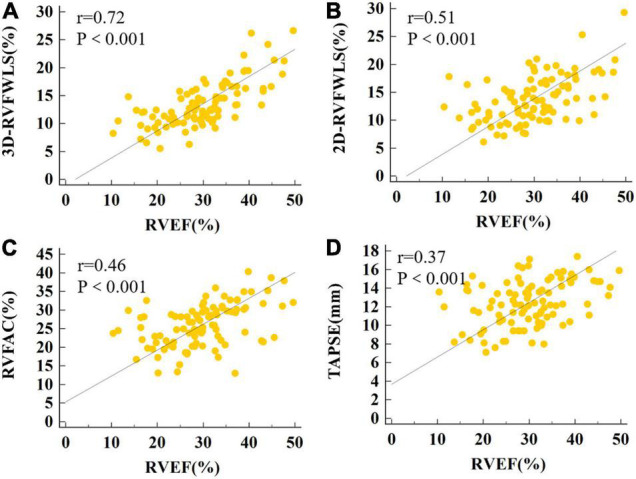
The correlations of RVEF with 3D-RVFWLS, 2D-RVFWLS and conventional echocardiographic parameters. The association between the 3D-RVFWLS **(A)**, 2D-RVFWLS **(B)**, RVFAC **(C)**, TAPSE **(D)**, and RVEF. 3D-RVFWLS and 2D-RVFWLS values are absolute values.

The relationships between RVEF, 2D- and 3D-STE, and conventional RV echocardiographic parameters are shown in Figure 5. RVEF was highly correlated with 3D-RVFWLS (*r* = 0.72, *P* < 0.001), modestly correlated with 2D-RVFWLS (*r* = 0.51, *P* < 0.001); and weakly associated with RVFAC (*r* = 0.46, *P* < 0.001), TAPSE (*r* = 0.37, *P* < 0.001), RVEDVI (*r* = –0.26, *P* = 0.017), RVESVI (*r* = –0.45, *P* < 0.001), and RVSV (*r* = 0.38, *P* < 0.001). Meanwhile, RVEF was not associated with RVD1, RVD2, RVD3, RIMP, or tricuspid s’. Moreover, 3D-RVFWLS correlated better with RVEF than 2D-RVFWLS and the conventional RV indices with RVEF (*r* = 0.72 vs. –0.26 to 0.51, *P* < 0.05). In addition, we found that CMR-RVEF was modestly correlated with 3D-RVFWLS (*r* = 0.53, *P* = 0.004), and weakly associated with 2D-RVFWLS (*r* = 0.49, *P* = 0.008) (Supplementary Figure 1).

### Reproducibility

The reproducibility of 2D- and 3D-STE parameters is shown in Table 3. 2D- and 3D-STE parameters had good intra- and inter-observer reproducibility, as evidenced by a higher ICC, small bias, and limits of agreement.

**TABLE 3:** Intraobserver and interobserver reproducibility.

Variables	ICC (95% CI)	Bias	Limits of agreement
**Intraobserver**			
RVEDV	0.98 (0.94–0.99)	4.2	–17.5, 25.9
RVESV	0.97 (0.93–0.99)	3.2	–14.7, 23.1
RVSV	0.93 (0.83–0.97)	–0.03	–12.2, 12.1
RVEF	0.89 (0.74–0.96)	–0.7	–7.8, 6.4
3D-RVFWLS	0.94 (0.84–0.97)	0.4	–1.8, 2.54
2D-RVFWLS	0.95 (0.88–0.98)	0.9	–0.9, 2.7
**Interobserver**			
RVEDV	0.94 (0.87–0.98)	6.8	–25.6, 39.2
RVESV	0.93 (0.84–0.97)	5.9	–21.9, 33.7
RVSV	0.92 (0.82–0.97)	0.8	–11.8, 13.5
RVEF	0.86 (0.68–0.94)	–0.6	–7.7, 6.6
3D-RVFWLS	0.91 (0.79–0.96)	0.5	–2.0, 3.0
2D- RVFWLS	0.90 (0.76–0.96)	0.8	–1.8, 3.3

*Abbreviations as in Table 1.*

## Discussion

To our knowledge, this may be the first investigation to comprehensively assess RV function in patients with ischemic and non-ischemic etiologies of end-stage HF using 2D- and 3D-STE and conventional echocardiographic parameters. The main findings of our study were as follows: (1) patients with ischemic or non-ischemic etiology of end-stage HF had diminished 2D- and 3D-RVFWLS compared to healthy controls; (2) patients with NICM had lower 3D-RVFWLS compared to ICM patients, although no significant difference in 2D-RVFWLS between these two subgroups was noted in our study; and (3) more importantly, ROC analysis revealed that 3D-RVFWLS displayed the potential for distinguishing NICM patients from ICM patients, while 2D-RVFWLS and conventional RV echocardiographic parameters did not. Therefore, 3D-STE may be superior to 2D-STE for distinguishing between the non-ischemic and ischemic etiologies of end-stage HF.

### RV Strain in Patients With End-Stage Heart Failure

Our results showed that 2D- and 3D-STE parameters were reduced in patients with ischemic or non-ischemic etiology of end-stage HF compared to healthy controls. These results are consistent with prior findings gathered using 2D-STE (20, 21). Our results have, for the first time, demonstrated that patients with end-stage HF have diminished 3D-RVFWLS. Several mechanisms may contribute to RV dysfunction in end-stage HF patients, including impaired LV function, elevated pulmonary arterial pressure, RV myocardial ischemia, and neurohormonal interactions (22). Patients with end-stage HF present with depressed LV function. Ventricular interaction could influence RV strain through the interventricular septum. RV failure in terms of histology includes rarefaction of myocardial capillaries and myocardial fibrosis (23, 24). Myocardial fibrosis results in myocardial remodeling and stiffness elevation, presenting with ventricular chamber enlargement and systolic dysfunction.

### RV Mechanics in Patients With Ischemic and Non-ischemic Etiologies of End-Stage Heart Failure

Differentiating NICM patients from ICM patients is essential on account of the different prognoses and treatment strategies for each group (18). Although the diagnosis is generally made by computed tomography angiography or coronary angiography, it is not practical for every patient to undergo an imaging study using angiography because of its radioactivity, invasiveness, and other contraindications. Therefore, it is critical to identify non-invasive parameters to distinguish between NICM and ICM. In our study, both algorithms provided substantially different results for patients with NICM and those with ICM, respectively. For example, we found that 3D-RVFWLS was lower in patients with NICM than those with ICM, but no difference in 2D-RVFWLS between the two subgroups was observed. With the use of 2D-STE algorithms, where speckles are only tracked in 2D planes, only a portion of the real myocardial motion is tracked. Thorstensen et al. reported 3D strain of left ventricle did not show incremental diagnostic value to the other modalities in patients with recent myocardial infarction, but patients with poor echocardiographic image quality were not excluded in their study and their study did not compare RV strain (25). A recent observation suggested that NICM exists as an intrinsic injury of the RV myocardium (21), and this proposal is compatible with our study findings revealed by 3D-STE. In contrast, in a small observational cohort of 40 patients with HF (including 20 with ICM and 20 with NICM), no significant difference in LVGLS obtained by 3D-STE between the ICM and NICM subgroups was noted (26). Another study, by Shanbhag et al., that followed a community-based sample of older adults for a median of 5.8 years showed that patients with NICM had a worse prognosis than those with ICM (6). Likewise, Meng et al. also demonstrated that patients with HF with poor clinical outcomes displayed impaired 3D-RVFWLS and RVEF compared to those without clinical events (27). Our findings provide direct evidence to support the aforementioned results. The fact that patients with NICM exhibited a poorer prognosis than those with ICM may be the reason why they also presented with more severely impaired 3D-RVFWLS. Moreover, ROC analysis revealed that the 3D-RVFWLS parameters had a good capacity for distinguishing NICM patients from ICM patients, while 2D-RVFWLS and conventional RV echocardiographic parameters failed to differentiate NICM patients from ICM patients. Therefore, 3D-RVFWLS may be a useful alternative to coronary angiography for distinguishing NICM from ICM, particularly among patients with end-stage HF who cannot undergo coronary angiography.

### Comparisons of Three-Dimensional-Speckle-Tracking Echocardiography and Two-Dimensional-Speckle-Tracking Strain Parameters

Owing to the complex geometry of the RV, 3D-STE has no geometric assumptions or out-of-plane motion of speckles, allowing for a more accurate assessment of myocardial performance by overcoming the limitations of 2D-STE. There were significant correlations between the RVFWLS values obtained by the 2D and 3D modalities. These findings were consistent with those of previous studies in patients with pulmonary hypertension (28). RV contraction occurs primarily in the form of longitudinal shortening (29). The longitudinal shortening of the RV free wall contributes to 80% of the RV stroke volume and may indicate the global RV function (30, 31). 2D-RVFWLS has been reported to exhibit prognostic value in various diseases, including HF (32–34). The good correlation and consistency of RVFWLS obtained by 2D- and 3D-STE suggest that 3D-STE may be a choice for RV function quantification and prognostic stratification.

### Correlations of RV Strain Parameters With RV Ejection Fraction

Our findings showed that there was a significant correlation between CMR-RVEF and 2D- and 3D-RVFWLS. This is compatible with findings of previous research, which demonstrated that CMR-RVEF was strongly related to 2D-RVFWLS (35). 3D echocardiography has been demonstrated to be more accurate than 2D echocardiography in the evaluation of RV function in patients with HF (36). RVEF as measured by 3D echocardiography has a great correlation with CMR-RVEF values (37) and has been considered a major determinant of RV systolic function in the updated 2015 recommendations (38). In this study, we also demonstrated that 3D-RVFWLS had a better association with RVEF than 2D-RVFWLS and conventional RV echocardiographic parameters.

### Clinical Implications

Unlike structurally normal hearts, patients with end-stage HF present with marked ventricular remodeling that may be better served by the 3D algorithm. Although 3D-STE has a theoretical superiority over 2D-STE for RV quantification, head-to-head comparisons between 3D-STE and 2D-STE for assessing RV strain in patients with end-stage HF have not yet been performed. Our results demonstrated the superiority of 3D-STE over 2D-STE and conventional RV echocardiographic indices in reflecting the RV myocardial pathophysiology in relation to the ischemic and non-ischemic etiologies of end-stage HF. Considering that 3D-STE better detects the changes in RV strain compared to 2D-STE in patients with ischemic and non-ischemic etiologies of end-stage HF, it should be the optimal choice for the assessment of RV strain in patients with end-stage HF. Additionally, we noted that 3D-RVFWLS was significantly related to 2D-RVFWLS and RVEF, and there were good consistency for RVFWLS as assessed by 2D- and 3D-STE, only 3D-RVFWLS can distinguish NICM patients from ICM patients, while 2D-RVFWLS and RVEF failed to distinguish these. Therefore, we suggest that 2D-STE and 3D-STE values should not be used interchangeably in patients with end-stage HF.

### Limitations

The present study had several limitations. First, 3D-STE analysis requires better image quality and experienced operators, and the technique is in its infancy and not yet widely validated for clinical use. Second, 3D-STE is hindered by low frame rates, which may have an effect on strain values. Third, we enrolled only patients with end-stage HF in this study, which may have led to a selection bias and an inability to generalize our findings to all patients with HF. Fourth, some patients with end-stage HF who were RV-paced or in a clinically critical state were not deemed suitable to undergo CMR examinations, so we could not obtain CMR data from all included patients. Fifth, although 3D-RVFWLS can distinguish NICM patients from ICM patients, while specificity of 50.5% is not so good. Sixth, 3D-STE parameters have vendor dependency (39); thus, our findings may not apply when using technology from other vendors. Ultimately, our study is a single-center observation with a relatively small number of patients. Future multicenter investigations with larger study populations are needed to confirm the superiority of 3D-STE over 2D-STE in quantifying RV performance in patients with end-stage HF.

## Conclusion

Our study demonstrated the superiority of 3D-RVFWLS over 2D-RVFWLS and conventional RV echocardiographic indices in identifying the ischemic and non-ischemic etiologies of end-stage HF. These findings indicate that 3D-RVFWLS may be a promising non-invasive imaging marker for distinguishing NICM from ICM.

## Data Availability Statement

The original contributions presented in the study are included in the article/Supplementary Material, further inquiries can be directed to the corresponding author/s.

## Ethics Statement

The studies involving human participants were reviewed and approved by the Ethics Committee of Tongji Medical College, Huazhong University of Science and Technology. The patients/participants provided their written informed consent to participate in this study.

## Author Contributions

FT, YG, YZ, BZ, YX, SY, JW, YY, QL, LZ, YML, and MX: conception and design of the study. FT, YG, YZ, YML, and MX: acquisition of data. MQ, YXL, SZ, YX, WW, SC, LZ, YML, and MX: analysis and interpretation of data. YML and MX: drafting the article. All authors contributed to the article and approved the submitted version.

## Conflict of Interest

The authors declare that the research was conducted in the absence of any commercial or financial relationships that could be construed as a potential conflict of interest.

## Publisher’s Note

All claims expressed in this article are solely those of the authors and do not necessarily represent those of their affiliated organizations, or those of the publisher, the editors and the reviewers. Any product that may be evaluated in this article, or claim that may be made by its manufacturer, is not guaranteed or endorsed by the publisher.

## Supplementary Material

Supplementary Figure 1 The Correlations of CMR-RVEF with RVFWLS. The association between the 2D-RVFWLS **(A)**, 3D-RVFWLS **(B)**, and CMR-RVEF.
