# Supplementary figures and images for: Evaluation of Right Ventricular Myocardial Mechanics by 2- and 3-Dimensional Speckle-Tracking Echocardiography in Patients With an Ischemic or Non-ischemic Etiology of End-Stage Heart Failure
Source: Front Cardiovasc Med. 2022 May 25;9:765191. doi: 10.3389/fcvm.2022.765191 (PMC9174453; doi:10.3389/fcvm.2022.765191)

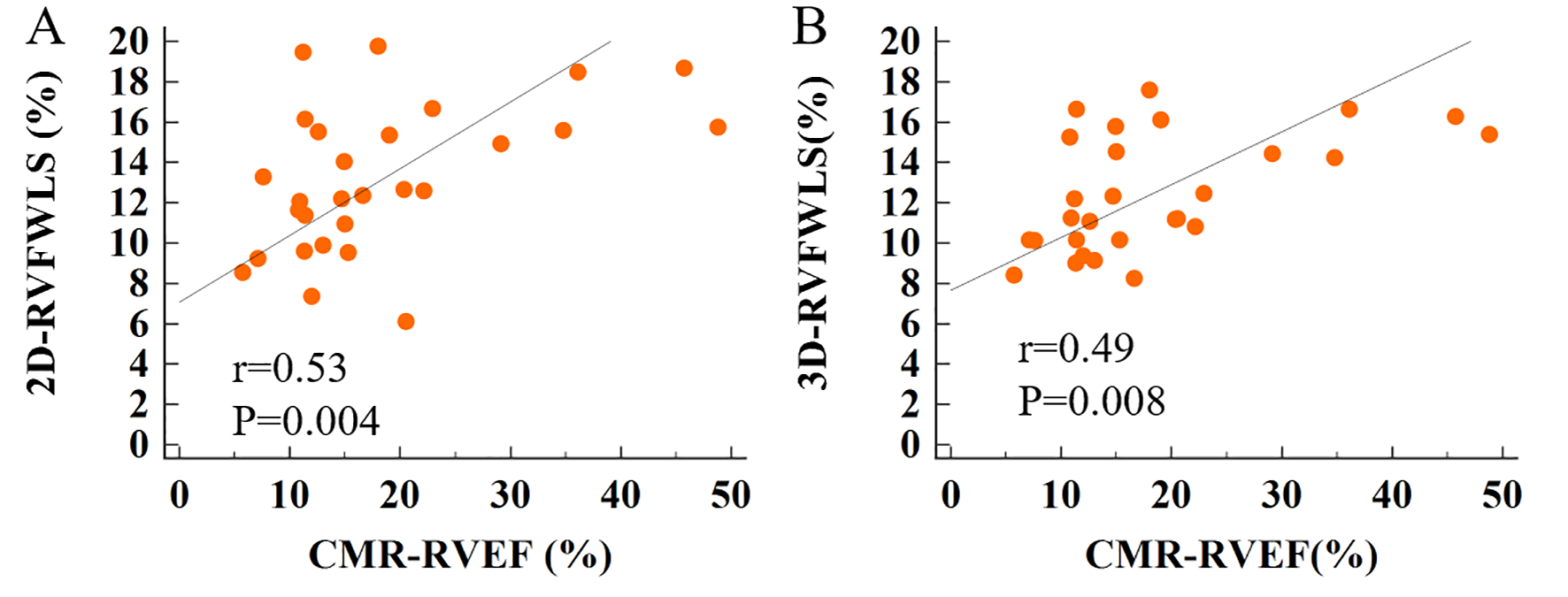

Supplement: Supplementary Figure 1 — The Correlations of CMR-RVEF with RVFWLS. The association between the 2D-RVFWLS (A), 3D-RVFWLS (B), and CMR-RVEF. [file Image_1.TIF]
